# Seizure Duration and Hemodynamic State during Electroconvulsive Therapy: Sodium Thiopental versus Propofol

**DOI:** 10.5539/gjhs.v8n2p126

**Published:** 2015-06-11

**Authors:** Hashem Jarineshin, Saeed Kashani, Fereydoon Fekrat, Majid Vatankhah, Javad Golmirzaei, Esmaeel Alimolaee, Hamid Zafarpour

**Affiliations:** 1 Anesthesiology, Critical Care and Pain Management Research Center, Hormozgan University of Medical Sciences, Bandar Abbas, Iran; 2 Research Center for Behavioral and Neurosciences, Hormozgan University of Medical Sciences, Bandar Abbas, Iran

**Keywords:** propofol, sodium thiopental, electroconvulsive therapy, psychiatric patients

## Abstract

**Introduction:**

General anesthesia is required for Electroconvulsive Therapy (ECT) and it is usually provided by a hypnotic agent. The seizure duration is important for the treatment, and it is usually accompanied by severe hemodynamic changes. The aim of this study was to compare the effects of sodium thiopental versus Propofol on seizure duration and hemodynamic variables during ECT.

**Methods:**

A number of 100 patient-sessions of ECT were included in this randomized clinical trial. The initial hemodynamic state of each patient was recorded. Anesthesia was induced by Sodium thiopental in the 1^st^ group and with Propofol in 2^nd^ group. All the patients received the muscle relaxant succinylcholine. The hemodynamic variables after seizure and seizure duration were recorded. The data were analyzed through SPSS 20 and independent t-test. P<0.05 was considered significant.

**Results:**

The mean duration of seizure in the sodium thiopental group was significantly longer than the Propofol group (40.3±16.6 sec versus 32±11.3 sec) (P=0.001). There was no statistically significant difference between the mean energy level applied in the two groups (20.5±3.81 joules in the sodium thiopental versus 20.2±3.49 joules in the Propofol group). The mean systolic and diastolic blood pressure at all times after seizure and mean heart rate at 3 and 5 minutes after seizure were significantly lower in Propofol than sodium thiopental groups.

**Discussion and Conclusion:**

Propofol provides a more stable hemodynamic state for the ECT procedures, and its use is highly preferred over sodium thiopental in patients with cardiovascular disease.

## 1. Introduction

ECT is considered one of the most effective treatments for major depression and unlike its alternative physical therapies, acts as a rapid-acting regimen for life-threatening psychiatric disorders. It continues to be a preferred modality among the modern treatments.The ECT treatment causes significant hemodynamic changes including tachycardia and hypertension which can be hazardous for some patients. The duration of seizure is the main defining factor for treatment success during ECT while the general anesthesia applied can attenuate its effectiveness (Miller & Pardo, 2011; Sadock, 2010). The ideal anesthetic medication for ECT should provide a rapid onset and recovery without altering the seizure duration while maintaining a stable hemodynamic state (Bauer, Hageman, Dam, Báez, Bolwig, & Roed, 2009; Dogan, Senoglu, Yildiz, Coskuner, & Ugur N, 2011; Patel, Gorst-Unsworth, Venn, Kelley & Yoav, 2006; Tan & Lee, 2009). For a long time, methohexital was the favorable anesthetic agent for ECT. However, reconsideration is needed nowadays to find alternative drugs, since methohexital has become rarely accessible in some countries (Ingram, Schweitzer, Ng, Saling, & Savage, 2007). A number of centers use sodium thiopental, which is a different drug from the barbiturates category, while in many other centers Propofolis widely used (Bauer et al., 2009; Dogan et al., 2011; Patel et al., 2006; Tan et al., 2009). Sodium thiopental usually does not have a rapid recovery, but Propofol provides a rapid onset and recovery even though it seemingly shortens the seizure duration and induces more hemodynamic changes as compared to sodium thiopental (Bauer et al., 2009; Dogan et al., 2011; Ingram et al., 2007; Patel et al., 2006; Tan et al., 2009). In a few medical centers, etomidate and inhalation anesthetics have also been used for this purpose in which neither drug demonstrated consistent effects in suppressing the rise in heart rate or blood pressure during ECT (Dogan et al., 2011; Tan & Lee, 2009). Patel et al., (2006) compared the effects of Etomidate to Propofol on ECT patients and showed that the Propofol grouprequired a double electricity dose for seizure induction and they recommended that a well organized randomly blinded study was needed to overcome their limitations.

The aim of this study was to evaluate the effects of sodium thiopental versus Propofol on seizure duration and hemodynamic parameters in patients during ECT.

## 2. Methods

### 2.1 Study Design

This study is a prospective double blind randomized clinical trial with a cross over design conducted on100 ECT patient-sessions. The patients were from 18 to 50 years of age with an ASA (American Society of Anesthesiologists) I status. In fact, a total of 50 patients with various psychiatric disorders who had undergone at least four ECT sessions were included in the study. Additionally, the patients who had undergone at least one ECT session met the inclusion criteria (i.e. entered the study at the second session onwards). Anesthesia for ECT in the first session was administered by a sodium thiopental or Propofol, while the second session 48 hours later involved another drug (cross-over).

### 2.2 Sample Estimation

In order to determine the sample size considering the previous studies (Bauer, Hageman, Dam, Báez, Bolwig, & Roed, 2009), that the mean seizure duration in the sodium thiopental and Propofol groups were 36.3 and 25.7 seconds respectively with a standard deviation of 10, an alpha of 0.05 and 80% power of statistical sample power analysis was applied, a number of 50 individuals in each group was calculated.

### 2.3 Ethics

The Research and Medical Ethics Committee of Hormozgan University of Medical Sciences approved the study project. Written consent was obtained of all the patients and their first-degree relatives.

### 2.4 Inclusion and Exclusion Criteria

Patients with a history of hypertension, diabetes mellitus, renal failure, neuromuscular and heart diseases were excluded.

### 2.5 Procedures

Non-invasive blood pressure, ECG, pulse oximetery (S/5 anesthesia monitor [Datex-Ohmeda, Finland]) and electroencephalogram-monitoring devices (Thymatron™ DGx Somatics INC. USA) were used for all patients. The baseline blood pressure, heart rate and peripheral oxygen saturation (SpO_2_) of all patients were measured and recorded. After pre-oxygenation, all the patients received 0.5 mg of intravenous atropine and were then divided randomly through block randomization into one of the following groups. The medications were specified by syringe A and B (the syringes were covered with a paper so that the contents were not visible to the researcher collecting the data), one of which was given to the patients in the first session, while in the second session at least 48 hours later the other syringe type which was not used for the related patient was administered.

The induction of anesthesia was done with either two anesthetic agents; 2-3 mg/kg intravenous sodium thiopental (syringe A) in the first group and 1-1.5 mg/kg intravenous Propofol (syringe B) in the second group. In addition, all the patients received the muscle relaxant succinylcholine at dose of 0.5 mg/kg. Ventilation with 100% oxygen was provided. The dental protection was provided by a bite block for all patients and then electrical stimulation was applied to induce seizure. The patients were ventilated with pure oxygen throughout the convulsion.

### 2.6 Statistical Analysis

The primary outcome in this study concerned the seizure duration. The secondary outcomes included the patient hemodynamic parameters such as systolic and diastolic blood pressure and heart rate and SpO_2_ which were recorded at different intervals after administering the drug including: before inducing seizure, immediately after seizure, 3 and 5 minutes after seizure. Similarly, the seizure duration was measured in two groups. The data were recorded in a special chart and then analyzed through SPSS 20, descriptive statistical measures and independent t-test, and P<0.05 was considered significant.

## 3. Results

In this clinical trial, the mean age of participants was 31.3±8.57 years old (Table 1).

**Table 1:** Frequency distribution of subjects under study sorted out by gender at different age groups

Age groups (Years old)	Gender	Frequency	Percentage
18-25	Male	**9**	**18**
	Female	**2**	**4**
26-38	Male	**19**	**38**
	Female	**13**	**26**
39-50	Male	**2**	**4**
	Female	**5**	**10**

Total	Male	**30**	**60**
	Female	**20**	**40**

The most common psychiatric disorder in the study was schizophrenia affecting 21 patients (42%), while the rarest one was obsessive-compulsive disorder affecting only 2 patients (4%). (Table 2)

**Table 2:** Frequency distribution of psychiatric disorders

No.	Diagnosis	Frequency	Percentage
**1**	Schizophrenia	**21**	**42**
**2**	Major depression Disorder	**5**	**10**
**3**	Bipolar Disorder	**12**	**24**
**4**	Obsessive-Compulsive Disorder	**2**	**4**
**5**	Other	**10**	**20**

	Total	**50**	**100**

There was no significant difference in the baseline and pre-ECT systolic blood pressure (SPB) and diastolic blood pressure (DBP) between two groups. However, the SPB and DBP levels after seizure were significantly lower in Propofol than the sodium thiopental group. (Table 3 and Table 4 respectively)

**Table 3:** Mean systolic blood pressures (mm Hg) in the two groups

Measurement Intervals	Sodium thiopental	Propofol	P-value
Baseline	119.16±10.68	117.64±11.42	0.224
Pre-ECT	121.90±10.89	120.02 ±11.42	0.197
Immediately after seizure	148.96±15.09	138.8 ±17.85	<0.001*
3 minutes after seizure	132.18±11.77	124.52 ±12.5	<0.001*
5 minutes after seizure	123.40±10.06	118.06±12.19	0.002*

* P<0.05 is significant.

**Table 4:** Mean diastolic blood pressures (mm Hg) in the two groups

Measurement Intervals	Sodium thiopental	Propofol	P-value
Baseline	75.52±7.89	75.08±8.61	0.737
Pre-ECT	78.18±7.94	76.74±8.88	0.226
Immediately after seizure	97.30±12.38	89.66±14.42	<0.001*
3 minutes after seizure	81.76±10.29	77.68±10.8	0.006*
5 minutes after seizure	76.78±9.13	73.36±9.58	0.009*

* P<0.05 is significant.

There was no significant difference in the heart rate (HR) between two groups at the times of baseline, pre-ECT and immediately after ECT but HR was significantly lower in the Propofol than sodium thiopental group at the 3 and 5 minutes after seizure. (Table 5)

**Table 5:** Mean heart rate (beat/min) in the two groups

Measurement Intervals	Sodium thiopental	Propofol	P-value
Baseline	88.3±19.20	87.32±20.7	0.588
Pre-ECT	90.28±23.7	90.7±24.1	0.807
Immediately after seizure	118.06±22.60	114.72±25.96	0.284
3 minutes after seizure	112.32±19.48	103.72±21.20	0.008*
5 minutes after seizure	108.74±17.31	104.56±16.78	0.031*

* P<0.05 is significant.

There was no statistically significant difference in the SpO_2_ between two groups at the baseline, before and after seizure. (Table 6)

**Table 6:** Mean of oxygen saturation rate (%) in the two groups

Measurement Intervals	Sodium thiopental	Propofol	P-value
Baseline	98 ±1.16	98.12±1.08	0.537
Pre-ECT	98.32±0.89	98.16±1.05	0.272
Immediately after seizure	99.24±1.02	99.3±0.83	0.690
3 minutes after seizure	99±1.19	98.88±1.27	0.595
5 minutes after seizure	98.94±1.01	98.92±1.14	0.919

*P<0.05 is significant.

There was no statistically significant different in the energy applied between two groups, but the seizure duration was significantly shorter in the Propofol group. (Table 7)

**Table 7:** The energy applied and the mean seizure duration in the two groups

Parameter measured	Sodium thiopental	Propofol	P-value
Energy applied (joule)	20.50±3.81	20.20 ±3.49	0.371
Seizure duration (sec)	40.32±16.67	32.02±11.36	0.001*

* P<0.05 is significant.

## 4. Discussion

In this study, the mean of seizure duration in the sodium thiopental group was longer than that in the Propofol group (40.3±16.6 sec versus 32±11.3 sec), which was statistically significant (P=0.001).

This finding is consistent with the results obtained from the other studies (Bauer et al., 2009; Ingram et al., 2007; Zaidi & Khan, 2000), which showed that the mean of seizure duration in the sodium thiopental-receiving group was significantly higher than that in the Propofol-receiving group. However, it was in contrast with the findings of a study by Kumar, Sharma and Mani (2012), where they concluded that the mean of seizure duration in the Propofol group was significantly (P<0.01) longer than that in the sodium thiopental group (94.45±21.37 sec versus 83±34.43 sec). This finding is probably due to the greater shock energy level applied to the patients in the Propofol group of their study. However, in our study, the mean of energy applied was nearly similar in the sodium thiopental and Propofol groups (20.5±3.8 and 20.2±3.4 joules, respectively). In the study done by Bauer et al. (2009) the energy applied for the sodium thiopental group and the Propofol group were 79.5 and 109.8 mC (mili-Coulombs), respectively that this can increase or decrease the seizure duration in the group which received higher or lower electrical charge.

In this study, the mean of baseline and pre-ECT SBP and DBP were similar in the both groups and these parameters were increased at time intervals after seizure but there was a significant increase of blood pressures in the sodium thiopental group as compared to the Propofol group.

These findings are consistent with those obtained by other researches (Omprakash, Ali, Anand, Devi, & Surender, 2008; Zaidi & Khan, 2000), which also showed that the mean of arterial blood pressure in the Propofol group was lower as compared to the sodium thiopental group.

Generally, the data collected in this study indicated no significant difference of baseline and pre-ECT hemodynamic parameters between the Propofol and sodium thiopental groups. Although there was an increase in these parameters of both groups immediately after seizure, but the increase in the heart rate at the third and fifth minute after seizure was less in propofol group compared to sodium thiopental group. This implies that the hemodynamic conditions in the Propofol group varied insignificantly before and after the ECT, which is consistent with the findings of Omprakash et al. (2008) and Zaidi and Khan (2000), who believed that Propofol provides a stable hemodynamic condition during ECT. Another interpretation to justify such findings is that, the common hemodynamic changes after ECT include bradycardia immediately followed by tachycardia and eventually hypertension and since Propofol causes more hypotension as compared to sodium thiopental, it can effectively prevent the subsequent ECT-induced hypertension (Miller et al., 2011; Sadock, 2010). Even some studies have demonstrated that induction of anesthesia with Propofol can further prevent hemodynamic responses of laryngoscopy and tracheal intubation (Jarineshin & Razmpour, 2006).

## 5. Conclusion

With regard to the above-mentioned findings and the favorable hemodynamic effects on patients in the Propofol group, we recommend the usage of Propofol for induction of anesthesia during ECT in patients with hypertension, hyperthyroidism, ischemic heart disease and other medical conditions in which the increase in hemodynamic parameters may be a threat for the patients’ well being. Although, the seizure duration was significantly shorter in the Propofol group as compared to sodium thiopental, the seizure duration above 25 seconds provides a dramatically effective therapy. Therefore, Propofol is generally considered a highly suitable drug for induction of anesthesia during ECT.

Our limitations are that we included patients who did not have any concurrent disease and if this was done in patients with underlying medical diseases, the results may have been different. We also did not evaluate the hemodynamic status and the overall profile of the patient during there covery phase. Further studies are needed to assess these issues and related patients’ outcomes.
